# Microsite and elevation zone effects on seed pilferage, germination, and seedling survival during early whitebark pine recruitment

**DOI:** 10.1002/ece3.3421

**Published:** 2017-09-25

**Authors:** Elizabeth R. Pansing, Diana F. Tomback, Michael B. Wunder, Joshua P. French, Aaron C. Wagner

**Affiliations:** ^1^ Department of Integrative Biology University of Colorado Denver Denver CO USA; ^2^ Department of Mathematical and Statistical Sciences University of Colorado Denver Denver CO USA

**Keywords:** microsite, *Pinus albicaulis*, recruitment limitations, regeneration, survival

## Abstract

Tree recruitment is a spatially structured process that may undergo change over time because of variation in postdispersal processes. We examined seed pilferage, seed germination, and seedling survival in whitebark pine to determine whether 1) microsite type alters the initial spatial pattern of seed caches, 2) higher abiotic stress (i.e. higher elevations) exacerbates spatial distribution changes, and 3) these postdispersal processes are spatially clustered. At two study areas, we created a seed distribution pattern by burying seed caches in microsite types frequently used by whitebark pine's avian seed disperser (Clark's nutcracker) in upper subalpine forest and at treeline, the latter characterized by high abiotic environmental stress. We monitored caches for two years for pilferage, germination, and seedling survival. Odds of pilferage (both study areas), germination (northern study area), and survival (southern study area) were higher at treeline relative to subalpine forest. At the southern study area, we found higher odds of 1) pilferage near rocks and trees relative to no object in subalpine forest, 2) germination near rocks relative to trees within both elevation zones, and 3) seedling survival near rocks and trees relative to no object at treeline. No microsite effects were detected at the northern study area. Findings indicated that the microsite distribution of seed caches changes with seed/seedling stage. Higher odds of seedling survival near rocks and trees were observed at treeline, suggesting abiotic stress may limit safe site availability, thereby shifting the spatial distribution toward protective microsites. Higher odds of pilferage at treeline, however, suggest rodents may limit treeline recruitment. Further, odds of pilferage were higher near rocks and trees relative to no object in subalpine forest but did not differ among microsites at treeline, suggesting pilferage can modulate the spatial structure of regeneration, a finding supported by limited clustering of postdispersal processes.

## 1 INTRODUCTION

Tree recruitment is a spatially structured process that shapes community development, population demographics, genetic structure, resiliency, and response to climate change (Aitken, Yeaman, Holliday, Wang, & Curtis‐McLane, 2008; Epperson & Chung, 2001; Holling, 1973; Hubbell, 2001; Nathan & Muller‐Landau, 2000). The initial spatial distribution of seeds on the landscape is determined by seed dispersal, and can remain unchanged over time or be altered by spatially variable postdispersal processes, including seed pilferage, seed germination, and seedling survival (Chambers & MacMahon, 1994; Gómez‐Aparicio, 2008; Houle, 1992; Jordano & Herrera, 1995; Rey & Alcántara, 2000; Schupp & Fuentes, 1995). In the former case, recruitment is spatially concordant, and thus, postdispersal processes are spatially consistent within heterogeneous landscapes. Spatial discordance occurs when the spatial distribution of seeds or seedlings differs from the original seed distribution, a result of seed or seedling survival rates varying with habitat and/or life stage (García, Ramón Obeso, & Martínez, 2005; Gómez‐Aparicio, 2008; Rother et al., 2015; Schupp & Fuentes, 1995). In addition, the seed distribution may be restructured if seeds are secondarily dispersed by abiotic mechanisms (e.g. runoff) or zoochory (e.g. Vander Wall, Kuhn, & Beck, 2005). Spatial discordance indicates recruitment is microsite‐limited, whereas concordance indicates seed limitation (Gómez‐Aparicio, 2008).

Spatial discordance occurs when postdispersal processes vary spatially or temporally (Chen, Liu, Chen, & Jia, 2012; García Ramón Obeso, & Martínez, 2005; Schupp, 1995; Schupp & Fuentes, 1995), and is often inferred via associations between survival and habitat type (García Ramón Obeso, & Martínez, 2005; Gómez‐Aparicio, 2008; Rother et al., 2015). Yet we know little about the causal mechanisms that shape the spatial distribution during early recruitment, and we are limited in our ability to connect seed dispersal to demographic consequences (Schupp & Fuentes, 1995; Wang & Smith, 2002). Experimental studies have examined regeneration niche characteristics and seedling distributions in herbaceous plant species by assessing survival of a single focal life stage or the consequences of seed limitations rather than microsite limitations (Turnbull, Crawley, & Rees, 2000 and references therein, Albrecht & McCarthy, 2009; Warren & Bradford, 2011). Experimental approaches in tree species have been limited to seed sowing or seedling transplant studies, which do not consider factors impacting seed survival and germination proclivity, respectively (e.g. Tomback et al., 2016).

Seed survival is largely limited by seed pilferage, yet we understand incompletely how seed pilferage affects recruitment spatial patterns. Andersen (1989) and Calviño‐Cancela (2007) suggest seed predation only influences recruitment in seed‐limited populations and therefore lacks spatial structure, whereas others suggest site‐specific conditions alter pilferage probability and create predictable spatial patterns (Pearson & Theimer, 2004; Vander Wall, 1998, 2000). Substrate type influences pilferage risk and rodent cache site selection behavior (Briggs & Vander Wall, 2004; Vander Wall, 1993), but the impacts of other microsite characteristics on pilferage risk and seed spatial distribution remain unknown.

Gómez‐Aparicio (2008) and Jara‐Guerrero, De la Cruz, Espinosa, Méndez, and Escudero (2015) suggest that the degree of spatial discordance may increase with abiotic stress because survival becomes restricted to fewer patches of suitable habitat. A natural gradient in environmental stress that provides an opportunity to assess these claims is the transition between subalpine forest and the alpine treeline ecotone (hereafter, “treeline”). Treeline, the transition from closed canopy subalpine forest to treeless alpine tundra, is characterized by higher wind speeds and UV exposure, and lower atmospheric pressure, temperatures, and growing season length relative to subalpine forests (Körner, 2012 and references therein). These stressors drive physiological changes across elevation gradients, including decreased cuticular respiration, and timing and duration of xylogenesis (Rossi, Deslauriers, Anfodillo, & Carraro, 2007; Rossi et al., 2008; Sowell, Koutnik, & Lansing, 1982). By examining postdispersal processes among different microsite types in subalpine forest and at treeline, we can assess the impact of abiotic environmental stress on spatial patterns of early recruitment. Here, we investigate whether microsite type and elevation zone lead to spatial discordance during early recruitment in whitebark pine (*Pinus albicaulis* Engelm. Pinaceae), a long‐lived upper subalpine and treeline conifer distributed throughout western North America.

Whitebark pine is a high elevation foundation and keystone species; it defines forest community structure and supports biodiversity (Ellison et al., 2005; Tomback & Achuff, 2010; Tomback, Arno, & Keane, 2001). Primary dispersal of whitebark pine seeds depends on a scatter‐hording bird, the Clark's nutcracker (*Nucifraga columbiana*, Corvidae), which is responsible for seed dispersal of several North American pines (Tomback & Linhart, 1990). Nutcrackers harvest seeds from indehiscent whitebark pine cones and bury them in caches throughout subalpine and treeline communities, returning to retrieve cached seeds during times of food scarcity (Hutchins & Lanner, 1982; Tomback, 1978, 1982; Tomback & Linhart, 1990). Caches not retrieved by nutcrackers may contribute to regeneration. This dispersal system is useful for experimental investigations of spatiotemporal recruitment dynamics because: (1) seeds are dispersed primarily by one mechanism (Hutchins & Lanner, 1982; Tomback, 1982), making the spatial distribution of caches simple to simulate experimentally; (2) large seeds (>0.1 g) allow single‐seed tracking; and (3) nutcracker caching is a major source of seedling recruitment in upper subalpine and treeline forests (Tomback, 1982, 1986). Natural recruitment in subalpine forest and at treeline provides two levels of environmental stress to assess spatial distribution changes associated with abiotic environmental conditions.

We hypothesized that (1) differential pilferage, germination, and/or seedling survival among microsite types, here defined by the type of object providing shelter to the seed cache or seedling, will alter the initial microsite distribution of seed caches, (2) higher abiotic stress (i.e. treeline relative to subalpine forest) will increase spatial discordance between consecutive life stages, and (3) successful postdispersal life stage transitions will be spatially clustered relative to failures. We predicted that (1) differential seed and seedling survival, resulting from pilferage, seed germination, and seedling survival, shifts the spatial distribution to microsites with better survival, and (2) microsite effects on postdispersal processes at treeline are more pronounced than in subalpine forest because of increased abiotic stress. In our study, we simulated Clark's nutcracker caches, placing them within specific microsite types known to be frequently used for seed dispersal. We focus on determining microsite and elevation zone effects on seed and seedling survival and their contribution to the general spatial distribution of regeneration. We do not attempt to predict the final spatial (microsite) distribution of seedlings because it will depend on site‐specific dynamics including seed availability and the initial microsite distribution of caches created by a population of nutcrackers.

## 2 METHODS

### 2.1 Study sites

**Figure 1:**
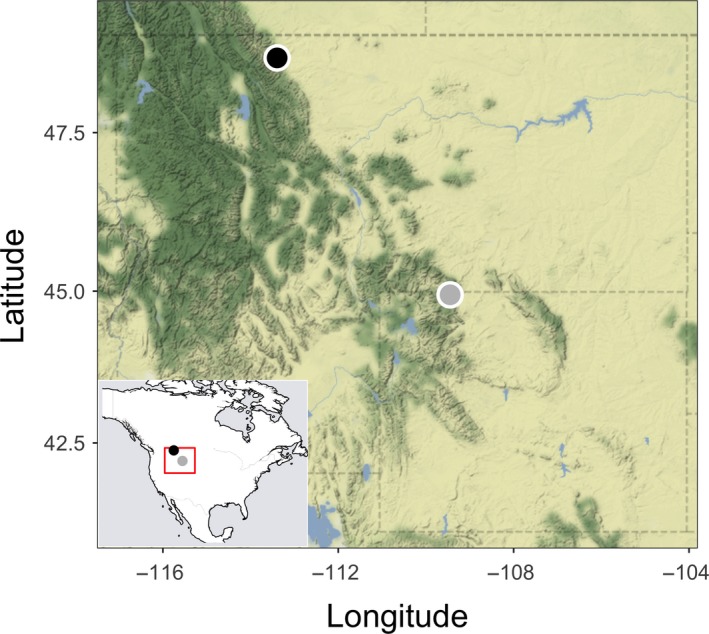
Study areas included White Calf Mountain, Glacier National Park, Montana (black), and Tibbs Butte, Shoshone National Forest, Wyoming (gray)

We conducted our study in two areas in the Rocky Mountains: Tibbs Butte, Shoshone National Forest, Wyoming (44° 56′ 28.33″N, 109° 26′ 39.69″W; 2,983–3,238 m elevation), and White Calf Mountain, Glacier National Park, Montana (48° 38′ 20. 95″N, 113° 24′ 08.72″W; 1,920–2,272 m elevation; Fig. 1). Both study areas include upper subalpine and treeline forest.

Soils on Tibbs Butte are shallow, coarse, and generally undeveloped, originating from Precambrian granite (Bevan, 1923; Nimlos & McConnell, 1965). During the study, air temperatures ranged from −30.7 to 16.2°C (July daily median = 11.9°C, February daily median = −9.10°C; PRISM Climate Group, 2015). Precipitation ranged from 0 to 51.6 mm/day (July daily median = 0.595 mm/day, February daily median = 3.29 mm/day; PRISM Climate Group, 2015). The subalpine forest comprises open late‐seral whitebark pine forest on south‐facing slopes and late‐seral mixed forest dominated by whitebark pine and Engelmann spruce (*Picea engelmannii* Parry ex Engelm.) on all other aspects. Dominant understory vegetation includes *Antennaria* spp., *Carex* spp., *Vaccinium scoparium* Leiberg ex Coville, and grasses. Solitary krummholz whitebark pine and Engelmann spruce dominate at treeline, with an understory matrix predominated by *Geum rossii* (R. Br.) Ser., *Potentilla diversifolia* Lehm., and *Saxifraga* spp.

White Calf Mountain soils belong to the Loberg‐Whitore association with exposed Altyn Formation white limestone (Lesica, 2002). Temperatures during the study ranged from −29.3 to 22.9°C (July daily median = 15.6°C, February daily median = −5.75°C; PRISM Climate Group, 2015), and precipitation ranged from 0 to 88.6 mm/day (July daily median = 0.650 mm/day, February daily median = 2.47; PRISM Climate Group, 2015). The subalpine forest comprises dense, closed canopy, late‐seral subalpine fir (*Abies lasiocarpa* (Hook.) Nutt.) forest, where whitebark pine is a minor component. Subalpine understory dominants include *Arnica cordifolia* Hook., *Arctostaphylos uva‐ursi* (L.) Spreng., *Pedicularis* spp., and *Thalictrum occidentale* A. Gray. At treeline, krummholz whitebark pine and subalpine fir dominate. Common understory species include *A. uva‐ursi, Achillea millefolium* L.*, Hedysarum sulphurescens* Rydb., *P. diversifolia*, and grasses.

### 2.2 Field methods

#### 2.2.1 Cache simulation

We created a cache distribution pattern at each study area, stratifying whitebark pine seed caches by elevation zone and microsite type as follows: In September 2011, we collected ripe whitebark pine seeds (McCaughey & Tomback, 2001) from Divide Mountain, Montana, and Line Creek Plateau Research Natural Area, Custer Gallatin National Forest, Montana, to cache on White Calf Mountain and Tibbs Butte, respectively. Seed sources were located no more than 11 km from their respective study sites, thus preserving local genetic structure (Mahalovich & Hipkins, 2011). We discarded unfilled (weight < 0.01 g), moldy, and/or pest‐infested seeds, simulating nutcracker seed selection behavior (Tomback, 1978, 1982; Vander Wall & Balda, 1977). We stored full seeds at ~1.5°C from November 2011 through June 2012 to simulate winter dormancy conditions necessary for whitebark pine seed germination (Burr, Eramian, & Eggleston, 2001; Tomback, Anderies, Carsey, Powell, & Mellmann‐Brown, 2001).

In early July through early August 2012, we created 372 caches over 51.0 hectares on Tibbs Butte and 362 caches over 47.8 hectares on White Calf Mountain. Caches at each study area were stratified by elevation zone and microsite type (Table 1). We separated elevation zones visually by evaluating an ESRI World Imagery satellite map to locate the boundary of contiguous closed canopy forest; we designated areas within closed canopy forest as subalpine and elevations above closed canopy forest as treeline (ESRI, 2012). We selected an equal number of point locations from each elevation zone at each study area using the random sampling tool in ArcGIS (ESRI, 2012). Caches were created in the three most common microsite types used as cache sites by Clark's nutcrackers: (1) at the base of trees (2) near rocks, and (3) in open areas near no object (Tomback, 1978, fig. 10). We systematically assigned one microsite type to each point location, creating a uniform distribution of seed caches among of microsite types. In the field, we navigated to each random point location and identified the closest assigned microsite type, where we created a cache. Caches created in rock or tree microsites were placed no more than 5 cm from the object (Vander Wall, 1982). We randomly selected the number of seeds per cache from a Poisson distribution (λ = 3, range = 1–7) created using cache‐size data of naturally occurring caches (data from Tomback, 1978 and Hutchins & Lanner, 1982). We did not systemically restrict the number of seeds per cache because we wanted to allow postdispersal processes to occur over the range of naturally existing cache sizes; the objectives of this study did not include assessing cache‐size effects on early recruitment. Seeds were buried at the average depth of nutcracker caches, ~2.5 cm (Tomback, 1982). Latex gloves were worn to limit the influence of human scent on cache pilferage (Duncan, Wenny, Spritzer, & Whelan, 2002; Vander Wall, 2000). We georeferenced all microsite locations (Trimble GeoXT GeoExplorer 2008 series), and triangulated exact cache locations using two nail spikes. Each nail spike was placed ~21 cm from the cache, with the cache representing the vertex of a 90‐degree angle. We left nail spikes in place to help locate caches in 2013 and 2014.

**Table 1:** Number of caches in each microsite type by elevation zone and study area that we located and assessed in 2013 (i.e. those used for analysis). The number of seeds indicates the total number of seeds sown in all caches of the specified microsite/elevation zone combination. Number of seeds per cache were randomly selected from a Poisson distribution (λ = 3, range = 1–7) generated using field based observations of nutcracker cache sizes (Hutchins & Lanner, 1982; Tomback, 1978)

Study area	Forest type	Microsite type	No. of caches created	No. of seeds cached
Tibbs Butte	Subalpine forest	Open	61	196
		Rock	58	180
		Tree	58	192
	Treeline	Open	64	242
		Rock	63	227
		Tree	62	227
White Calf Mountain	Subalpine forest	Open	58	226
		Rock	59	209
		Tree	58	211
	Treeline	Open	51	162
		Rock	67	230
		Tree	58	193

#### 2.2.2 Cache assessment

In late July and early August of 2013 and 2014, we revisited caches made in 2012 to determine the number of individual seeds remaining (2013 only), first‐year seedlings, and one‐year‐old seedlings (2014 only). In 2013, we counted the number of first‐year seedlings, missing seeds, and seed coats (evidence of rodent predation) at each cache (McCaughey, 1994; Mirov, 1967). We considered caches to have germinated if one or more seeds in the cache germinated, producing first‐year seedlings. The number of missing seeds was determined by excavating each cache without disturbing seedlings. We assumed all missing seeds were pilfered by small granivorous rodents, which rely on olfactory cues to locate caches (Vander Wall, 1998). We assumed no seed loss from nutcrackers or other avian seed predators, because these species rely primarily on visual cues and memory, and generally locate their own caches (Vander Wall, 1982). Intact seeds were repositioned in the same cache. In 2014, we returned to caches that contained intact seeds or first‐year seedlings in 2013 and estimated survival and second‐year seed germination, as delayed germination is common in whitebark pine (Tomback, et al., 2001). Caches examined in 2014 could contain either one‐year‐old seedlings (i.e. germinated in 2013) or first‐year seedlings (i.e. germinated in 2014); we identified second‐year seedlings by the presence of fascicular (adult) needle growth. We did not assess second‐year pilferage because soil compaction, potentially from freeze‐thaw processes, increased substantially between 2013 and 2014. Excavating seeds under these conditions could kill seedlings, thereby impacting our ability to continue monitoring seedling survival in the future.

### 2.3 Analysis

#### 2.3.1 Assessing differences in postdispersal processes between elevation zones and among microsite types

We estimated odds ratios and corresponding 95% confidence intervals (CIs) to quantify the dissimilarity in odds of pilferage, germination, and seedling survival between study areas, among microsite types, and between elevation zones (Rita & Komonen, 2008). Odds ratios were estimated for all pairwise comparisons (i.e. Tibbs Butte and White Calf Mountain, rock and tree, rock and no object, no object and tree, and treeline and subalpine forest). We used the cache as our sampling unit because whitebark pine seedling clusters often fuse to form one tree (Linhart & Tomback, 1985; Tomback & Linhart, 1990), and it maintains the assumption of statistical independence required for data analysis, as seeds in the same cache experience similar conditions. Odds were calculated using the formula $\left(\frac{p}{1-p}\right)$, where *p* represents the proportion of caches with one or more pilfered seeds, one or more germinated seeds, or one or more surviving seedlings. Odds ratios were calculated as a ratio of the odds, $\left(\frac{{\text{Odds}}_{\mathrm{Group}\;1}}{{\text{Odds}}_{\mathrm{Group}\;2}}\right)$. For example, if the proportion of caches that germinated at one study site was 0.25, the proportion of caches failing to germinate would be 0.75. The resulting odds of germination would be 0.25/0.75 or 0.333, meaning the probability of cache germination for the first study site is 1/3 the probability of nongermination. If the odds of germination were 0.15 at a second study site, the odds ratio for germination at the first study site relative to the second would be 0.333/0.15 or 2.22, indicating the odds of germination at the first study site are 2.22 times larger than the odds of germination at the second study site. In contrast, an odds ratio of 1.0—the “null” model—occurs when there are equal odds of germination and nongermination between study sites. If 95% CIs included an odds ratio of 1.0, we concluded no difference between the odds of the events of interest occurring. Sample sizes for each life stage differed because cache germination and survival depended on successful transition from the previous stage. We analyzed pilferage using the total number of caches we located in 2013, germination using the number of caches that had one or more intact seeds following pilferage and/or first‐year seedlings in 2013, and seedling survival using the number of caches with one or more first‐year seedlings in 2013. All analyses were conducted in R (version 3.2.3, R Core Team, 2015).

#### 2.3.2 Spatial clustering of postdispersal processes

We estimated the difference in Ripley's *K* function to determine whether (1) pilferage was clustered relative to seed survival, (2) germination was clustered relative to nongermination, and (3) seedling survival was clustered relative to seedling mortality, and the scales at which clustering occurred.

Ripley's *K* function uses point to point distances to describe distribution patterns and determine whether points are clustered, uniform, or randomly distributed and the scale of pattern occurrence (Ripley, 1977). This process has been extended to assess the relative distribution of marked points by estimating the difference between Ripley's *K* functions generated for each type of marked point. We considered *successes* to have occurred at caches that were pilfered, had first‐year seedlings in 2013, or second‐year seedlings in 2014. We assumed cache sites were Poisson point processes governed by the same spatial intensities up to a proportionality constant (Bivand, Pebesma, & Gómez‐Rubio, 2013). For each study area and elevation zone combination, we compared the difference in estimated *K* functions for the observed data with the estimated difference in *K* functions for 1,000 data sets simulated under the random labeling hypothesis, where the probability of success is equal for all caches (Diggle, 2014). For each simulated data set, caches were randomly assigned as a success or failure in the same proportion as the original data. Simulated data sets were used to construct 95% confidence envelopes for the true difference in *K* functions under the random labeling hypothesis. Distances at which the difference in estimated *K* functions exceeds the upper limit of the confidence envelope suggest clustering of successes relative to failures at that spatial scale, whereas the difference falling below the lower limit of the confidence envelope suggests clustering of failures relative to successes at that spatial scale. (French, 2015; R Core Team, 2015).

## 3 RESULTS

### 3.1 Pilferage, germination, and survival

**Figure 2:**
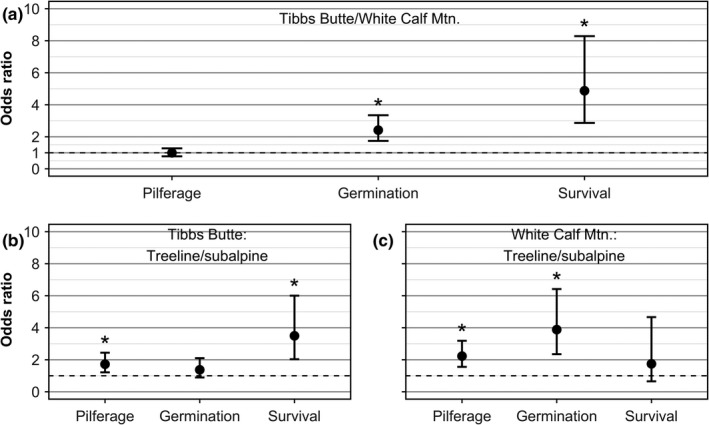
Odds ratio (OR) estimates and 95% CIs comparing odds of cache pilferage, germination, and survival of (a) Tibbs Butte relative to White Calf Mountain, and treeline relative to subalpine forest on (b) Tibbs Butte and (c) White Calf Mountain. Dotted line indicates the null model of no difference in survival between variable levels (OR = 1.0). Pairwise comparisons whose CIs do not overlap 1.0 suggest odds of success in the first group are higher than those in the second

In 2013, we found 717 of the original 735 caches. Rodents pilfered at least one seed from 54.3% of all cache sites; 74.9% of the pilfered caches lost all seeds, and 25.1% lost only a portion of seeds originally cached. We found seed coats showing evidence of immediate consumption of seeds near 65.0% (*n* = 197) and 22.0% (*n* = 189) of pilfered caches on Tibbs Butte and White Calf Mountain, respectively. Odds of pilferage did not differ between study sites; rodents pilfered 53.8% of the caches at each site (Fig. 2a, Tables 2 and 3). However, odds of germination and survival were 2.42 (95% CI: 1.57, 3.70) and 4.87 (95% CI: 2.87, 8.28) times higher, respectively, on Tibbs Butte than White Calf Mountain (Fig. 2a). On Tibbs Butte, at least one seed germinated in 64.1% of caches with seeds remaining after pilferage (*n* = 217; Table 2), whereas on White Calf Mountain, at least one seed germinated in 42.5% of caches with surviving seeds (*n* = 212; Table 3). On Tibbs Butte and White Calf Mountain, respectively, at least one‐first‐year seedling in 62.6% (*n* = 139; Table 2) and 25.6% (*n* = 90; Table 3) of caches with seedlings in 2013 survived to 2014.

**Table 2:** Percent and 95% confidence intervals of caches with one or more pilfered seeds, germinated seeds, and seedlings that survived on Tibbs Butte, Shoshone National Forest, Wyoming, in total, by elevation zone, and by microsite type

Study area	Percent pilfered^a^ 95% CI	Percent germinated^b^ 95% CI	Percent survival^c^ 95% CI	Elevation zone	Percent pilfered^a^ 95% CI	Percent germinated^b^ 95% CI	Percent survival^c^ 95% CI	Microsite	Percent pilfered^a^ 95% CI	Percent germinated^b^ 95% CI	Percent survival^c^ 95% CI
Tibbs Butte	53.8 48.6–59.0 (*n* = 366)	64.1 57.2–70.4 (*n* = 217)	62.6 53.9–70.5 (*n* = 139)	Treeline	60.3 52.9–67.3 (*n* = 189)	68.0 57.7–76.9 (*n* = 97)	77.3 65.0–86.3 (*n* = 66)	Rock	60.3 47.2–72.2 (*n* = 63)	77.4 58.5–89.7 (*n* = 31)	87.5 66.5–96.7 (*n* = 24)
								No object	57.8 44.8–69.8 (*n* = 64)	70.0 53.3–82.9 (*n* = 40)	60.7 40.7–77.9 (*n* = 28)
								Tree	62.9 49.7–74.6 (*n* = 62)	53.8 33.7–72.9 (*n* = 26)	92.9 64.2–99.6 (*n* = 14)
				Subalpine forest	46.9 39.4–54.5 (*n* = 177)	60.8 51.5–69.5 (*n* = 120)	49.3 37.5–61.2 (*n* = 73)	Rock	56.9 43.3–69.6 (*n* = 58)	73.7 56.6–86.0 (*n* = 38)	42.9 25.0–62.6 (*n* = 28)
								No object	31.1 20.2–44.4 (*n* = 61)	59.2 44.3–72.7 (*n* = 49)	51.7 32.9–70.1 (*n* = 29)
								Tree	53.4 40.0–66.5 (*n* = 58)	48.5 31.2–66.1 (*n* = 33)	56.3 30.6–79.2 (*n* = 16)

a Percent of caches that lost ≥1 seeds between July 2012 and July 2013.

b Percent of caches with ≥1 seeds remaining after pilferage that germinated in 2013.

c Percent of caches with ≥1 living seedlings in 2013 that survived until July 2014.

**Table 3:** Percent and 95% confidence intervals of caches with one or more pilfered seeds, germinated seeds, and seedlings that survived on White Calf Mountain, Glacier National Park, Montana, in total, by elevation zone, and by microsite type

Study area	Percent pilfered^a^ 95% CI	Percent germinated^b^ 95% CI	Percent survival^c^ 95% CI	Elevation zone	Percent pilfered^a^ 95% CI	Percent germinated^b^ 95% CI	Percent survival^c^ 95% CI	Microsite	Percent pilfered^a^ 95% CI	Percent germinated^b^ 95% CI	Percent survival^c^ 95% CI
White Calf Mountain	53.8 48.5–59.1 (*n* = 351)	42.5 35.8–49.4 (*n* = 212)	25.6 17.2–36.0 (*n* = 90)	Treeline	63.6 56.0–70.6 (*n* = 176)	61.4 50.3–71.4 (*n* = 88)	29.6 18.4–43.8 (*n* = 54)	Rock	62.7 50.0–73.9 (*n* = 67)	62.9 44.9–78.0 (*n* = 35)	27.3 11.6–50.4 (*n* = 22)
								No object	58.8 44.2–72.1 (*n* = 51)	58.1 39.3–74.9 (*n* = 31)	44.4 22.4–68.7 (*n* = 18)
								Tree	69.0 55.3–80.1 (*n* = 58)	63.6 40.8–82.0 (*n* = 22)	14.3 2.5–43.8 (*n* = 14)
				Subalpine forest	44.0 36.6–51.7 (*n* = 175)	29.0 21.4–38.0 (*n* = 124)	19.4 8.8–36.6 (*n* = 36)	Rock	40.7 28.3–54.2 (*n* = 59)	32.6 19.5–48.7 (*n* = 43)	21.4 5.7–51.2 (*n* = 14)
								No object	44.8 32.0–58.4 (*n* = 58)	25.0 13.2–41.5 (*n* = 40)	30.0 8.1–64.6 (*n* = 10)
								Tree	46.6 33.5–60.0 (*n* = 58)	29.3 16.6–45.7 (*n* = 41)	8.3 0.4–40.2 (*n* = 12)

a Percent of caches that lost ≥1 seeds between July 2012 and July 2013.

b Percent of caches with ≥1 seeds remaining after pilferage that germinated in 2013.

c Percent of caches with ≥1 living seedlings in 2013 that survived until July 2014.

### 3.2 Microsite effects

**Figure 3:**
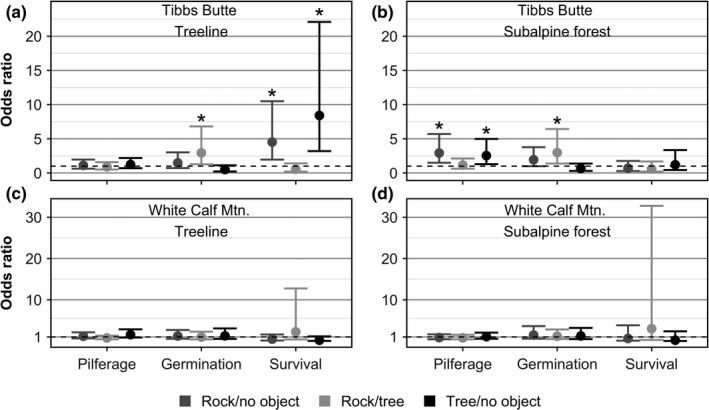
Odds ratio (OR) estimates and 95% CIs comparing odds of cache pilferage, germination, and survival by microsite type on Tibbs Butte (a, b) and White Calf Mountain (c, d). Rock/no object indicates comparison of odds of success for rocks relative to no object; Rock/tree compares odds of rocks relative to trees; Tree/no object compares trees relative to no object. Dotted line indicates the null model of no difference in survival between variable levels (OR = 1.0). Pairwise comparisons whose CIs do not overlap 1.0 suggest odds of success in the first group are higher than those in the second

At treeline on Tibbs Butte, odds of pilferage did not differ among microsite types (Fig. 3a). Odds of germination were 2.94 (95% CI: 1.27, 6.81) times higher near rocks than trees, and similar between rocks and trees relative to no object. Odds of seedling survival were 4.53 (95% CI: 1.95,10.5) and 8.41 (95% CI: 3.20, 22.1) times greater near rocks and trees, respectively, relative to no object. Odds of seedling survival were similar between rocks and trees (Fig. 3a).

In subalpine forest on Tibbs Butte, odds of pilferage near rocks and trees were 2.92 (95% CI: 1.49, 5.70) and 2.58 (95% CI: 1.29, 4.98) times higher than near no object, respectively (Fig. 2). Odds of one or more seeds germinating were 2.98 (95% CI: 1.08, 8.20) times higher near rocks than trees, but odds of germination did not differ when comparing rocks and trees to no object. Odds of seedling survival were similar among microsite types (Fig. 3b). The proportion of caches that were pilfered, germinated, and survived by microsite type on Tibbs Butte is shown in Table 2.

On White Calf Mountain, no differences in the odds of pilferage, germination, or seedling survival by microsite type were detected in either elevation zone (Fig. 3c,d). The proportion of caches that were pilfered, germinated, and survived by microsite type on White Calf Mountain is shown in Table 3.

### 3.3 Elevation zone effects

On Tibbs Butte, odds of pilferage were 1.72 (95% CI: 1.21, 2.44) times higher at treeline than in subalpine forest (Fig. 2b). Rodents pilfered one or more seeds from 60.3% of treeline and 46.9% of subalpine forest caches. Odds of germination in caches containing seeds that survived pilferage did not differ between elevation zones (Fig. 2b). At least one seed germinated in 68.0% of treeline caches and 60.8% of subalpine forest caches (Table 2). Odds of seedling survival at treeline on Tibbs Butte were 3.49 (95% CI: 2.03, 6.00) times higher than odds of survival in the subalpine forest (Fig. 2b). At treeline and in subalpine forest, respectively, 77.3% and 49.3% of caches with one or more first‐year seedlings in 2013 survived to 2014 (Table 2).

On White Calf Mountain, odds of pilferage were 2.23 (95% CI: 1.56, 3.19) times higher at treeline than in subalpine forest (Fig. 2c). Rodents pilfered 63.6% of treeline caches and 44.0% of subalpine caches (Table 3). Odds of germination at treeline were 3.88 (95% CI: 2.35, 6.42) times higher than in the subalpine forest (Fig. 2c). One or more seeds in caches with seeds remaining germinated in 61.4% of treeline and 29.0% of subalpine caches (Table 3). Odds of survival did not differ between elevation zones (Fig. 2c). At treeline and in the subalpine forest, respectively, 29.6% and 19.4% of first‐year seedlings survived from 2013 to 2014 (Table 2).

### 3.4 Additional regeneration resulting from delayed germination

Between cache placement in 2012 and mid‐July 2014, 65.8% of caches with seeds surviving pilferage germinated. Delayed germination (i.e. cache germination after our 2013 measurements) occurred in 35.9% and 0.66% of caches on Tibbs Butte and White Calf Mountain, respectively (Table 4). Thirty‐nine caches had seeds that germinated asynchronously (i.e. seeds in the same cache germinated in both 2013 and 2014).

**Table 4:** Percent and 95% confidence intervals of caches that germinated in 2014, and 2013 and 2014 combined. The total number of caches represents those with seeds remaining following pilferage (Tibbs Butte: *n* = 217, White Calf Mountain: *n* = 212)

Study area	Percent germinated 2014^a^ 95% CI	Percent germinated 2013 & 2014^b^ 95% CI	Elevation zone	Percent germinated 2014^a^ 95% CI	Percent germinated 2013 & 2014^b^ 95% CI	Microsite	Percent germinated 2014^a^ 95% CI	Percent germinated 2013 & 2014^b^ 95% CI
Tibbs Butte	35.9 29.6–42.8	84.8 79.2–89.2	Treeline	44.3 34.4–54.8	89.7 81.4–94.7	Rock	32.3 17.3–51.5	90.3 73.1–97.5
						No object	40.0 25.3–56.6	90.0 75.4–96.7
						Tree	65.4 44.4–82.1	88.5 68.7–97.0
			Subalpine forest	29.2 21.4–38.3	80.8 72.4–87.2	Rock	26.3 14.0–43.4	92.1 77.5–97.9
						No object	30.6 18.7–45.6	79.6 65.2–89.3
						Tree	30.3 16.2–48.9	69.7 51.1–83.8
White Calf Mountain	6.6 3.8–11.1	46.2 39.4–53.2	Treeline	6.8 2.8–14.8	64.8 53.8–74.5	Rock	5.7 1.0–20.5	62.9 44.9–78.0
						No object	6.5 1.1–22.8	61.3 42.3–77.6
						Tree	9.1 1.6–30.6	72.7 49.6–88.4
			Subalpine forest	6.5 3.0–12.7	33.1 25.0–42.2	Rock	7.0 1.8–20.1	37.2 23.4–53.3
						No object	10.0 3.3–24.6	30.0 17.1–46.7
						Tree	2.4 0.1–14.4	31.7 18.6–48.2

a Percent of caches with ≥1 intact seeds in 2013 that germinated in 2014.

b Percent of all caches with ≥1 unpilfered seeds that germinated in either 2013 or 2014.

Of the caches containing intact seeds in 2013, 84.8% on Tibbs Butte and 46.2% on White Calf Mountain germinated in either 2013 or 2014 (Table 4). Odds of having germinated after 2 years were 6.49 (95% CI: 4.76, 8.84) times higher on Tibbs Butte than White Calf Mountain. At treeline on Tibbs Butte, odds of germination in 2013 or 2014 were 2.21 (95% CI: 1.31, 3.72) times higher than in subalpine forest. In subalpine forest, odds of germination near rocks were 3.11 (95% CI: 1.20, 8.08) times higher than near trees, with no differences between other microsite pairs. On White Calf Mountain, odds of germination in 2013 or 2014 at treeline were 3.87 (95% CI: 1.95, 7.66) higher than in subalpine forest. No differences in odds of germination by microsite type were detected at treeline on Tibbs Butte or in either forest type on White Calf Mountain.

In 2014, living seedlings were found at 35.8% (95% CI: 30.9, 41.0) and 7.1% (95% CI: 4.8, 10.5) of the 717 caches found in 2013 on Tibbs Butte and White Calf Mountain, respectively.

### 3.5 Spatial patterns of postdispersal processes

At treeline on Tibbs Butte, the difference in *K* function exceeded the upper boundary of the confidence envelope at distances greater than 110 m, indicating pilfered cache locations are clustered relative to unpilfered cache locations at scales greater than 110 m. We found no evidence of a difference in relative clustering in the subalpine forest at Tibbs Butte. In both elevation zones on White Calf Mountain, we detected clustering of pilfered caches relative to unpilfered caches at 34 m (Fig. S1). Comparing the caches that germinated in 2013 to the locations where no seeds germinated, we detected clustering of cache locations that failed to germinate relative to those that successfully germinated at distances of 17–25 m at treeline on Tibbs Butte. No clustering of germinated caches was detected in subalpine forest on Tibbs Butte. We detected clustering of cache locations that germinated relative to cache locations that failed to germinate on White Calf Mountain at scales of 80–100 m at treeline and from 17 to 18 m in subalpine forest (Fig. S2).

On Tibbs Butte, no clustering patterns of caches containing surviving seedlings were detected at treeline. In subalpine forest, caches with surviving seedlings were clustered from 31 to 39 m, at 53 m, and from 61 to 64 m in subalpine forest. On White Calf Mountain, mortality was clustered relative to survival from 9 to 11 m at treeline. In subalpine forest, survival was clustered from 92 to 93 m, whereas survival was clustered from 38 to 42 m (Fig. S3).

## 4 DISCUSSION

### 4.1 Microsite effects on whitebark pine recruitment

We found that the microsite distribution of caches changed over time on Tibbs Butte, and the nature of these changes differed by elevation zone. In contrast, on White Calf Mountain, no microsite effects were detected in either elevation zone. Our results suggest that (1) the microsite distribution of caches can be altered by postdispersal processes, leading to spatial discordance between life stages (e.g. the proportion of caches near rocks will be higher following germination than following seed pilferage), (2) whitebark pine recruitment limitations may vary by location, and (3) safe sites vary by life stage. Our results on Tibbs Butte suggest that whitebark pine recruitment can be microsite‐limited even when suitable microsite types remain on the landscape. “Availability” of suitable microsite types is determined by the frequency of nutcrackers caching in specific microsite types. Microsite limitation does not exclude the possibility that seed limitation may also restrict recruitment (Clark et al., 1999).

Lack of microsite effects and lower overall recruitment success on White Calf Mountain suggests that environmental conditions, such as substrate, light availability, and local climate, must be suitable for recruitment before microsite effects appear, and/or safe sites may be more important in some locations than others. Variation in annual weather, densities of small granivorous rodents, seed/pollen availability, and Clark's nutcracker occurrence or caching behavior may influence recruitment success at larger spatial scales (Crone, McIntire, & Brodie, 2011; Rapp, McIntire, & Crone, 2013). Consequently, we cannot generalize the recruitment process and its variability across spatial scales; patterns must be examined locally.

Microsite types that promote regeneration can vary with postdispersal process. In the subalpine forest, for example, odds of cache pilferage were higher near objects, whereas the odds of germination were higher near objects, suggesting sites with lower pilferage risk are distinct from those that promote germination. Gómez‐Aparicio (2008) define safe sites as those that promote survival during the highest risk life stages. Pilferage was the main recruitment bottleneck (40% seed loss) in this study. Because microsites near protective objects had higher odds of pilferage, caches near no object may be safe sites for whitebark pine recruitment on Tibbs Butte.

### 4.2 Treeline recruitment

We predicted that increased abiotic stress at treeline would result in more pronounced changes to the microsite distribution of caches than observed in subalpine forest, leading to spatial discordance during postdispersal processes. At treeline on Tibbs Butte, odds of seedling survival were higher in microsites near trees and rocks relative to those near no object, indicating a distributional shift of caches toward microsites near objects. In contrast, there was no difference in microsite distribution of caches between first‐ and second‐year seedlings in subalpine forest. This supported our hypothesis that higher abiotic stress (i.e. treeline relative to subalpine forest) increased spatial discordance between life stages.

Higher odds of survival near trees and rocks at treeline on Tibbs Butte may be a result of competition shifting to facilitation as abiotic stress increases (Bertness & Callaway, 1994; Brooker et al., 2008; Callaway, 1998; Callaway, Brooker, Choler, & Kikvidze, 2002; Pyatt et al., 2016). At treeline, both rocks and trees reduce wind speed, and trees reduce exposure to photosynthetically active radiation (Pyatt et al., 2016). Similar facilitative interactions occur in other northern Rocky Mountain treelines and on Tibbs Butte specifically, where mature trees are most frequently found near rocks (Resler & Tomback, 2008; Tomback, Chipman, Resler, Smith‐McKenna, & Smith, 2014; Tomback et al., 2016). This suggests microsite effects during early recruitment influence the spatial pattern of mature treeline forests and may persist through tree maturity.

Odds of germination (White Calf Mountain) and survival (Tibbs Butte) were higher at treeline than in subalpine forest, indicating that germination and first‐year seedling survival are not limiting recruitment at treeline at either study area; this suggests that recruitment may be responding to climate change. Millar, Westfall, Delany, Flint, and Flint (2015) suggest that recent increases in synchronous pine recruitment at treeline are moderated by climate variables including water relations and temperature. However, if we assume pilfered seeds are lost to the recruitment pool, potential recruitment increases may be offset by increased odds of pilferage at treeline, limiting the rate of treeline advance in response to climate change (Munier et al., 2010). These findings reinforce the importance of considering cache pilferage in plant regeneration studies; if cache pilferage were not monitored, we would likely not conclude that seed germination or seedling survival were higher at treeline.

### 4.3 Spatial structure of pilferage

Microsite effects during cache pilferage differed between elevation zones, altering the original cache distribution on Tibbs Butte. Higher odds of pilferage at treeline at both study areas shifted the distribution toward subalpine caches. Higher odds of pilferage near trees and rocks in subalpine forest on Tibbs Butte changed the spatial distribution of caches, with more caches remaining near no object. In contrast, cache pilferage at treeline did not vary by microsite type.

Observed spatial structuring of pilferage in subalpine forest could result if rodents better detect caches in specific microsite types. For example, Vander Wall (2010) showed that *Tamias amoenus* and *Peromyscus maniculatus*, the most common granivorous rodent species at our study areas (Pansing, 2014), found seed caches with higher water content more frequently, but soil moisture was unlikely to vary with microsite type (Pyatt et al., 2016). Instead, higher odds of pilferage near objects may be determined by cache encounter rates, as rodents forage near cover to decrease predation risk (Barnum, Manville, Tester, & Carmen, 1992; Fanson, 2010; Stapp & Van Horne, 1997; Thayer & Vander Wall, 2005).

Pilferage was spatially structured in subalpine forest but lacked spatial structure at treeline on Tibbs Butte. This may be because rodents at higher elevations require more food and larger home ranges (Desy, Batzli, & Liu, 1990; Hammond, Roth, Janes, & Dohm, 1999; Quirici et al., 2010), meaning rodents may move more widely at treeline. This could lead to higher encounter rates of seed caches and explain the lack of microsite preference for cache pilferage at treeline.

Pilfered caches were spatially clustered relative to unpilfered caches at distances larger than 110 m at treeline on Tibbs Butte, and from 33 to 34 m in both elevation zones on White Calf Mountain. Clustering may result from spatial heterogeneity in rodent distribution, home range size, substrate type, vegetation distribution, or other abiotic characteristics (Briggs & Vander Wall, 2004). Although spatial clustering has been used to describe how rodents cache seeds (Puerta‐Piñero, Gómez, & Schupp, 2010), we are unaware of studies assessing spatial patterns in pilferage.

### 4.4 Seed predation or secondary dispersal

Forty percent of seeds in this study were removed from the recruitment pool by seed pilferage. Our conclusions about seed loss and changes to the spatial distribution of whitebark pine seed caches resulting from pilferage assume that every pilfered seed was consumed. Vander Wall, Kuhn, & Beck, (2005) caution against associating pilferage with predation, because pilfered seeds can be secondarily dispersed (Greene & Johnson, 1997; Vander Wall, Kuhn, & Beck, 2005). It is possible that some seeds in both study areas were recached by rodents, which could alter the original cache distribution pattern. However, we found seed coats with signs of predation near 65% and 22% of pilfered caches on Tibbs Butte and White Calf Mountain, respectively. In addition, we tracked rodent‐dispersed seeds to address this question (Pansing, 2014): Over three nights in July 2013, at White Calf Mountain, we tracked fluorescent pigment‐coated whitebark pine seeds provided at seed stations, following the methods of Longland and Clements (1995) and Tomback, Schoettle, Chevalier, and Jones (2005). We detected seven track lines leading to surface caches containing 1–3 seeds and eight lines ending in burrows. These observations suggest that pilfered seeds are surface cached or larder‐hoarded. Neither fate leads to seed germination (Levitt, 1972; Tomback, Schoettle, Chevalier, & Jones, 2005). Although this method precluded assessing the caching behavior of diurnal *Tamias* spp., fewer than 1% of individuals trapped at the study area belonged to this genus; the remaining species (*P. maniculatus* and *Zapus princeps*) were nocturnal (Pansing, 2014).

If we consider the scenario that all pilfered seeds were secondarily dispersed to the best microsite type for germination (e.g. rocks on Tibbs Butte), we would expect the first‐year seedling distribution to shift more strongly toward rock microsites than we observed. However, rodents cache most often under shrubs and trees to reduce predation risk (Thayer & Vander Wall, 2005; Vander Wall & Joyner, 1998). On Tibbs Butte, there are few shrubs, and secondary seed dispersal by rodents might be directed toward trees, which have lower odds of germination. Thus, even if most pilfered seeds were secondarily dispersed, this might not greatly influence the spatial pattern of seed germination and seedling survival.

### 4.5 Limitations to this study

Whitebark pine is a masting species, characterized by intermittent, synchronous, high magnitude cone production (Crone, McIntire, & Brodie, 2011; Tomback, 1982). The predator satiation hypothesis predicts that mast years overwhelm seed predators and decrease the proportion of predated seeds (Kelly & Sork, 2002; Silvertown, 1980). Thus, seed dispersal, and ultimately recruitment, may be highest following a mast year. Whether a mast year would alter the spatial structure of recruitment would depend on whether the postdispersal processes of seed pilferage, germination, and seedling survival retain the same spatial distribution that we observed during this study. Studies during a mast year would allow us to test this question.

Our methodological approach assessed changes to the microsite and elevation zone distributions of early life stages of whitebark pine. However, we did not test specific conditions that increase life stage‐specific odds of survival. Although microsite and elevation zone characteristics vary (e.g. Körner, 2012; Pyatt et al., 2016), more research is needed into the specific drivers of differential survival, such as microclimate conditions or competition with neighboring vegetation.

Clark's nutcrackers cache across a range of possible microsite types and elevation zones, but are thought to prefer specific microsite characteristics for seed caching, including proximity to trees and increased understory cover (Lorenz, Sullivan, Bakian, & Aubrey, 2011; Tomback, 1982). Because we sought to determine the cache sites that result in increased survival, we created a microsite distribution of caches stratified by elevation zone and microsite type. This allowed us to assess whether these features influenced the odds of seed survival, germination, and seedling survival, but not to predict the final distribution of recruitment across microsites. We found that microsite type and elevation zone can *cause* differential survival and therefore alter regeneration distribution relative to previous life stages.

Because rodents respond to visual and olfactory cues and nutcrackers respond to visual cues, seeds may have been pilfered by individuals who associated human scent or nail spikes with seed caches (Duncan, Wenny, Spritzer, & Whelan 2002). To reduce these effects, we wore gloves when creating caches, and did not cache when nutcrackers or other birds were near. Ultimately, we detected differences in odds of pilferage between elevation zones and among microsite types, suggesting we did not greatly influence pilferage probability. Further, limited clustering of pilfered relative to unpilfered caches suggests that cues did not influence the pattern of pilferage. If learning did occur, we would expect greater clustering of cache pilferage, as one animal pilfered all nearby caches.

## CONFLICT OF INTEREST

The authors declare that they have no conflict of interest.

## AUTHOR CONTRIBUTIONS

ERP, DFT, and MBW designed the experiment. ERP, ACW, and DFT collected data. ERP, MBW, and JPF performed statistical analyses. ERP, DFT, MBW, ACW, and JPD wrote the manuscript. All authors have reviewed and approved the final manuscript.
