# Supplementary material for: Microsite and elevation zone effects on seed pilferage, germination, and seedling survival during early whitebark pine recruitment
Source: Ecol Evol. 2017 Sep 25;7(21):9027–40. doi: 10.1002/ece3.3421 (PMC5677468; doi:10.1002/ece3.3421)

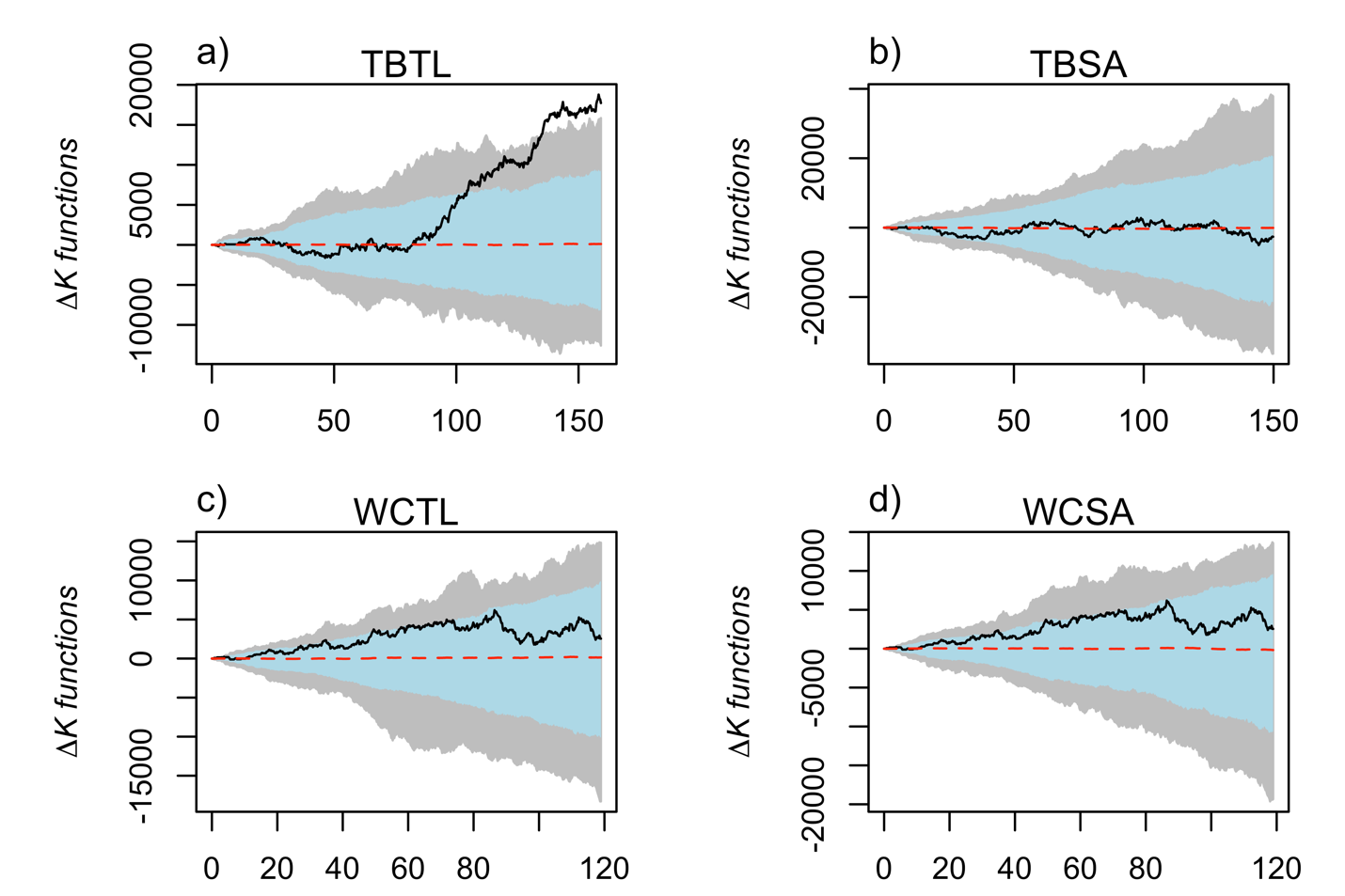
Figure S1: Difference in Ripley’s K functions between caches with one or more pilfered seeds in 2013 relative to caches without pilfered seeds in each elevation zone at each study area. Gray shading represents the most extreme difference from 499 randomly labeled data sets. Light blue shading represents 95% confidence bands for the true difference in K functions. Note that distance scales differ between plots based on maximum distance between caches. TBTL: Tibbs Butte treeline; TBSA: Tibbs Butte subalpine; WCTL: White Calf Mountain treeline; WCSA: White Calf Mountain subalpine.


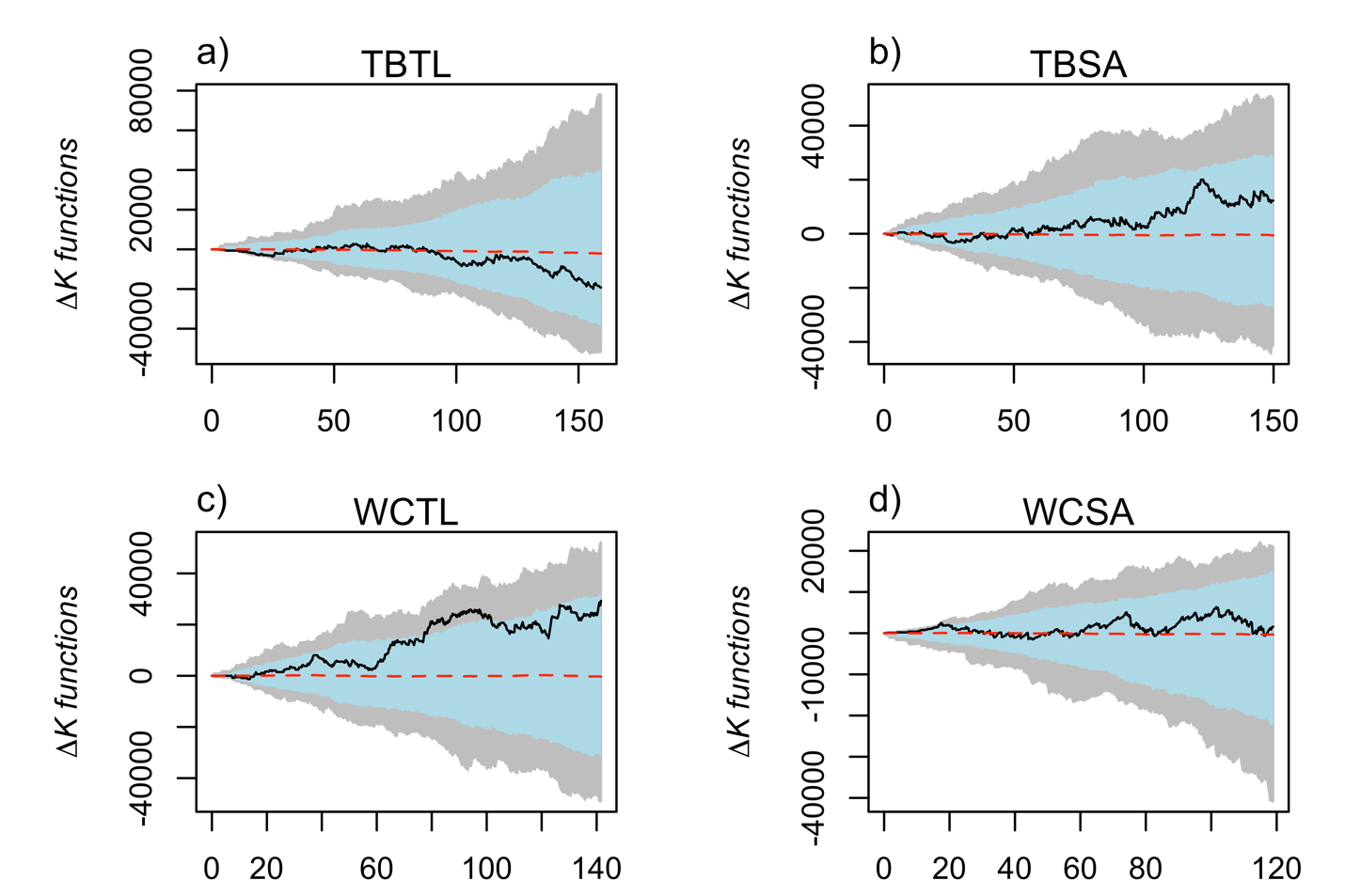
Figure S2: Difference in Ripley’s K functions between caches with one or more seeds that germinated in 2013 and those that did not germinate in each elevation zone at each study area. Gray shading represents the most extreme difference from 499 randomly labeled data sets. Light blue shading represents 95% confidence bands for the true difference in K functions. Note that distance scales differ between plots based on maximum distance between caches. TBTL: Tibbs Butte treeline; TBSA: Tibbs Butte subalpine; WCTL: White Calf Mountain treeline; WCSA: White Calf Mountain subalpine.

Figure S3: Difference in Ripley’s K functions between caches with seedlings that survived from August 2013 to August 2014 and those that died during the same time period in each elevation zone at each study area. Gray shading represents the most extreme difference from 499 randomly labeled data sets. Light blue shading represents 95% confidence bands for the true difference in K functions. Note that distance scales differ between plots based on maximum distance between caches. TBTL: Tibbs Butte treeline; TBSA: Tibbs Butte subalpine; WCTL: White Calf Mountain treeline; WCSA: White Calf Mountain subalpine.


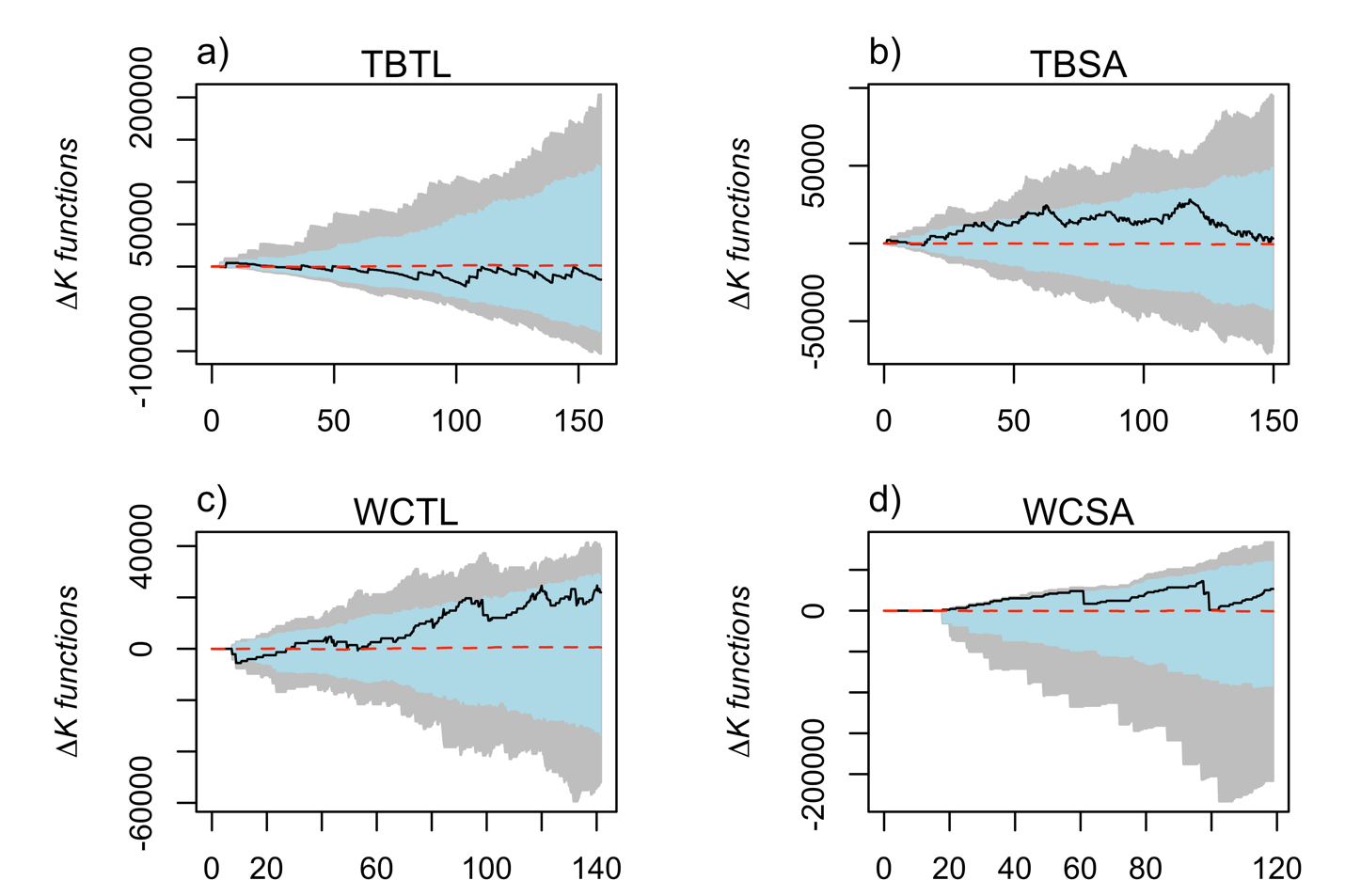

Supplement: Supplementary file 1 [file ECE3-7-9027-s001.docx]
